# Differential Oxygenation in Tumor Microenvironment Modulates Macrophage and Cancer Cell Crosstalk: Novel Experimental Setting and Proof of Concept

**DOI:** 10.3389/fonc.2019.00043

**Published:** 2019-02-06

**Authors:** Noelia Campillo, Bryan Falcones, Jordi Otero, Roser Colina, David Gozal, Daniel Navajas, Ramon Farré, Isaac Almendros

**Affiliations:** ^1^Unitat de Biofísica i Bioenginyeria, Facultat de Medicina i Ciències de la Salut, Universitat de Barcelona, Barcelona, Spain; ^2^CIBER de Enfermedades Respiratorias, Madrid, Spain; ^3^Department of Child Health, University of Missouri-School of Medicine, Columbia, MO, United States; ^4^Institute for Bioengineering of Catalonia, The Barcelona Institute of Science and Technology, Barcelona, Spain; ^5^Institut d'Investigacions Biomèdiques August Pi i Sunyer, Barcelona, Spain

**Keywords:** hypoxia gradient, macrophage motility, models of host-tumor interactions, novel assay technology, tumor progression

## Abstract

Hypoxia is a common characteristic of many solid tumors that has been associated with tumor aggressiveness. Limited diffusion of oxygen generates a gradient of oxygen availability from the blood vessel to the interstitial space and may underlie the recruitment of macrophages fostering cancer progression. However, the available data based on the recruitment of circulating cells to the tumor microenvironment has been so far carried out by conventional co-culture systems which ignore the hypoxic gradient between the vessel to the tumor interstitium. Here, we have designed a novel easy-to-build cell culture device that enables evaluation of cellular cross-talk and cell migration while they are being simultaneously exposed to different oxygenation environments. As a proof-of-concept of the potential role of differential oxygenation among interacting cells we have evaluated the activation and recruitment of macrophages in response to hypoxic melanoma, breast, and kidney cancer cells. We found that hypoxic melanoma and breast cancer cells co-cultured with normoxic macrophages enhanced their directional migration. By contrast, hypoxic kidney cells were not able to increase their recruitment. We also identified well-described hypoxia-induced pathways which could contribute in the immune cell recruitment (VEGFA and PTGS2 genes). Moreover, melanoma and breast cancer increased their proliferation. However, oxygenation levels affected neither kidney cancer cell proliferation nor gene expression, which in turn resulted in no significant changes in macrophage migration and polarization. Therefore, the cell culture device presented here provides an excellent opportunity for researchers to reproduce the *in vivo* hypoxic gradients in solid tumors and to study their role in recruiting circulating cells to the tumor in specific types of cancer.

## Introduction

Cancer progression in solid tumors is characterized by rapid cellular growth along with alterations in the stromal microenvironment all of which foster invasion and spreading to distant organs. Hypoxia is now recognized as a frequent and important feature of rapidly growing and aggressive solid tumors and is the result of the imbalance between the supply and consumption of oxygen in the tumor. This imbalance is primarily caused by the high metabolic rate of tumor cells in conjunction with the presence of aberrant angiogenesis and disorganized and leaky vascularization (1, 2). In these settings, primary solid tumors usually present with maladaptive blood flow supply, such that greater distances between tumor cells and vessels become apparent (1, 3), with the resultant low values of oxygen partial pressure (<0.1–5%) in those regions leading to regional tumor necrosis (3, 4). In contrast, the tumor periphery where the tumor is advancing and invading is better irrigated by newly developed vessels (1, 5). In the context of the high metabolic rate of tumor cells and the limited diffusion of oxygen in the interstitial space, a distance gradient of oxygen availability emerges between the blood vessel and adjoining tumor cells. For instance, it has been reported that at a distance of 200 μm from the vessel the oxygen supply is drastically reduced (6).

**Figure 1:**
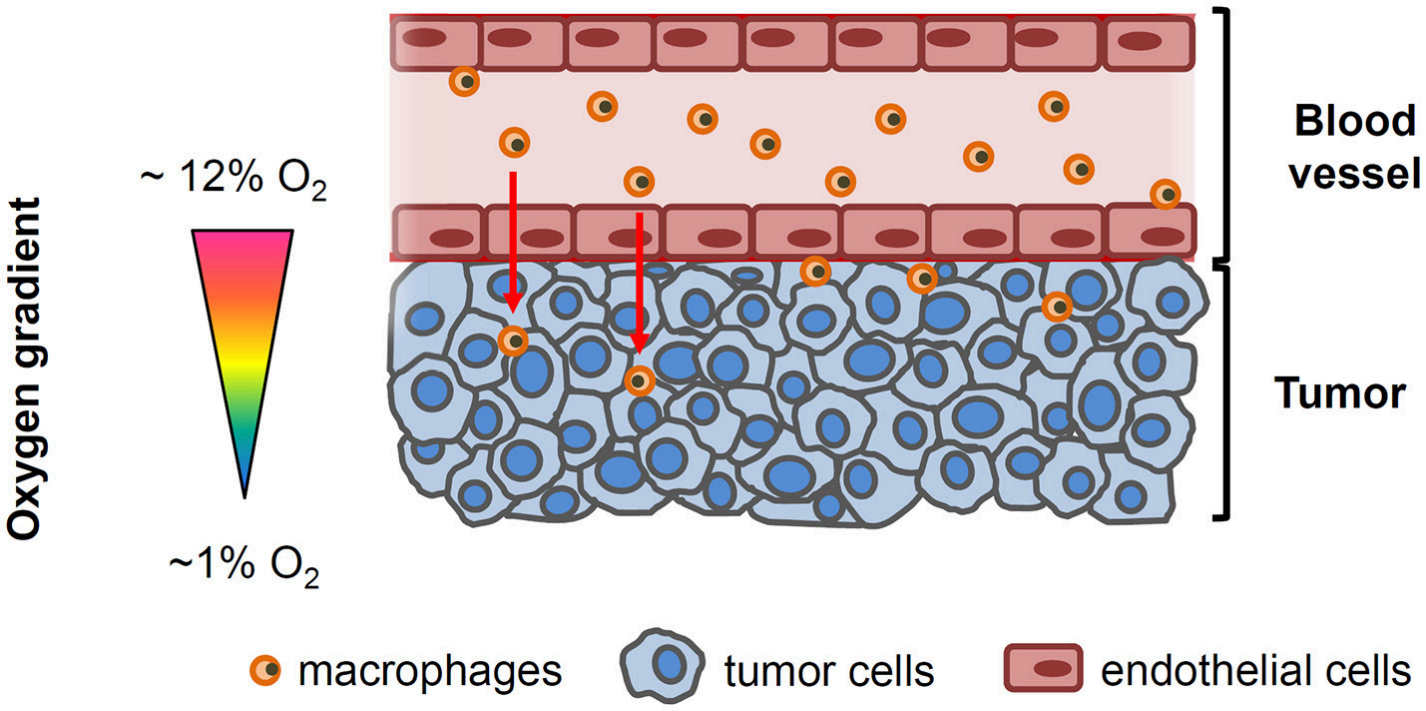
Simplified drawing showing the oxygenation gradient from the blood vessel to tumor cells. Circulating macrophages and endothelial cells lining the tumor vessel are exposed to the physiological oxygen concentrations in blood (~12 O_2_) while oxygen levels within the tumor decline as the distance from the functional blood vessel increases.

There is increasing evidence linking tumor hypoxia with poor cancer prognosis (2, 6–8). One of the mechanisms through which hypoxia could enhance the tumor malignant properties is by inducing changes in the immune system that facilitate tumor escape from immunosurveillance (2). As part of the orchestrated response of the tumor, it has become apparent that macrophages, the most abundant immune cell population in the majority of solid tumors, play a critical role at each stage of cancer progression (7). Human and experimental studies have consistently demonstrated that macrophages are preferentially recruited to tumors with greater and more extensive hypoxic regions, and that hypoxia-induced changes in macrophages can also enhance cancer malignancy (9) (Figure 1). Furthermore, a recent *in vitro* study has shown that tumor cells pre-exposed to hypoxia enhanced the attraction of macrophages toward the tumor (10). However, notwithstanding the aforementioned evidence, the available *in vitro* models that are used to characterize and reproduce the crosstalk between cancer cells and host immune cells do not incorporate in their characteristics the ability to recapitulate the oxygen gradient that exists from the vessel to the tumor interstitium. In fact, most studies are usually carried out using conventional migration and invasion assays in the presence of a homogeneously oxygenated environment (usually at room air oxygenation). Therefore, the potential role of the O_2_ gradient on macrophage recruitment, as well as other circulating cells, is poorly explored and understood (11). To address this issue and to further our insights on the role of oxygen gradients in solid tumor progression, we developed a novel experimental setting that is capable of studying the crosstalk between two cell types, with each of these cells being concurrently subjected to different and well-controlled oxygen partial pressures. This system further enabled the creation of differential oxygen environments between the bottom (hypoxia) and upper (normoxia) compartments of a conventional transwell porous membrane mimicking the higher oxygen environments that are present in close proximity to the vascular network and the hypoxic levels in other more distant regions where tumor cells are continuously exposed to poor oxygenation (Figure 1). As proof-of-concept of this model we also investigated whether differential oxygen levels mimicking those in the pathophysiologic conditions that characterize solid tumors could facilitate macrophage recruitment to the tumor and further promote increased tumor cell proliferation.

## Materials and Methods

### Cell Culture and Reagents

Mouse macrophages (RAW264.7), mouse melanoma (B16F10), and mouse renal adenocarcinoma (RENCA) cell lines were purchased from the American Type Culture Collection (ATCC, Manassas, VA). Murine breast cancer cells (E0771) were obtained from CH3 BioSystems (Buffalo, NY). All cell lines were routinely grown in high glucose Dulbecco's modified Eagle's medium (DMEM) supplemented with 10% fetal bovine serum (Gibco, Gaithersburg, MD), and an antibiotic/antimycotic solution at final concentrations of 100 U/ml penicillin, 100 μg/ml streptomycin and 0.250 μg/ml amphotericin B (Sigma-Aldrich, St. Louis, MO). Cells were maintained in T-25 tissue culture flasks (Techno Plastic Products AG, Trasadingen, Switzerland) in a standard humidified incubator with 5% CO_2_ balanced-air at 37°C. Prior to experimentation, RAW264.7 cells were serum deprived. Fluorescence-conjugated antibodies purchased from BioLegend (San Diego, CA) were employed to identify macrophages (CD45-FITC, clone30-F11) and to determine their phenotype (CD86-PerCP/Cy5.5 -clone GL-1- for M1 phenotype and CD206-APC -clone C08C02- for M2 phenotype) by fluorescence-activated cell sorting (FACS).

### Co-culture Model to Study the Crosstalk of Cells Exposed to Different Oxygen Environments

**Figure 2:**
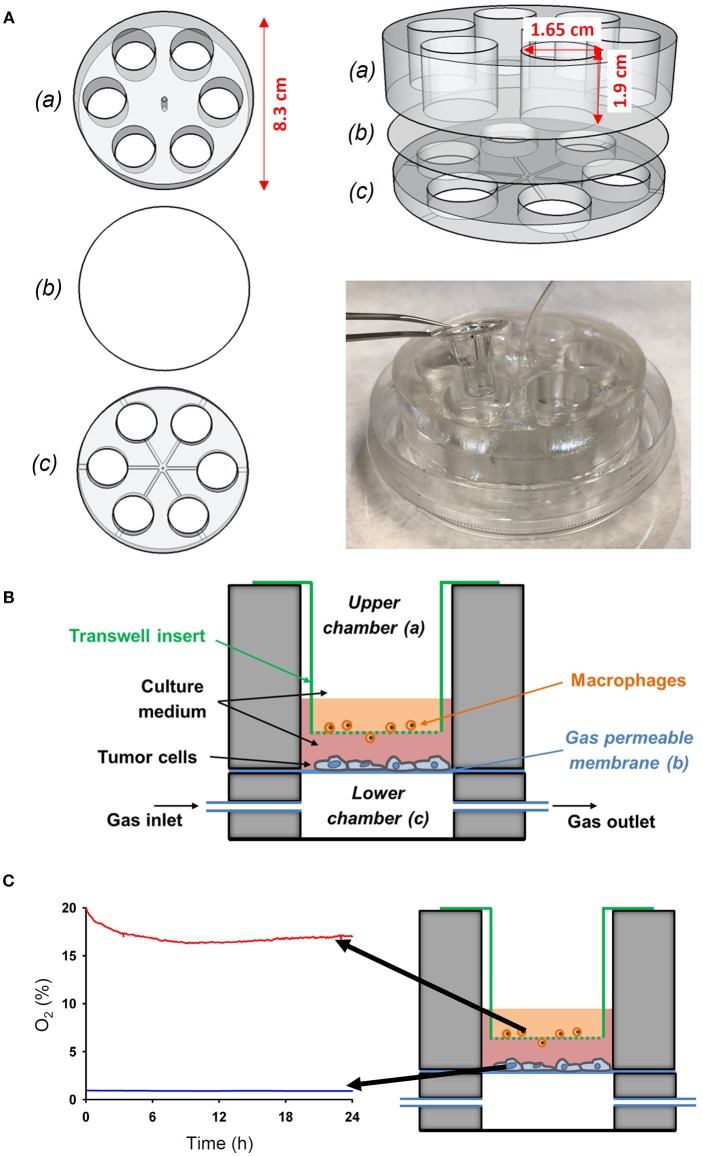
Novel experimental *in vitro* setting to reproduce different oxygenation patterns between the tumor-vessel interface. **(A)** The device was performed by assembling an upper (a) and a lower (c) chamber with a PDMS membrane (b) between both chambers. The wells of the upper chamber where adjusted to fit to a conventional transwell insert. **(B)** Scheme of the novel *in vitro* experimental setting developed to study the interactions between tumor cells and macrophages simultaneously exposed to different oxygen levels mimicking the pathophysiological conditions in the solid tumor microenvironment. **(C)** The novel setting was able to produce an oxygen gradient as measured by an oxygen optical sensor.

A custom-made co-culture system based on a thin membrane permeable to oxygen was designed to investigate the interaction between cells simultaneously subjected to differential oxygen microenvironment. The device consists of two polydimethylsiloxane (PDMS) well layers separated by a 37.5 μm thick commercially available membrane (Gel-Pak, Hayward, CA) (Figure 2). The membrane constitutes a gas permeable substrate for cell culture. Oxygen concentration over the cell culture can be tightly controlled via direct diffusion through the gas permeable membrane from a gas source (Gas Blender 100 Series, MCQ Instruments, Rome, Italy) connected to the lower layer. More specifically, the time required for the gas stimulus to diffuse through the Gel-Pak membrane was ~5 s as previously determined (12). The setting was designed to allow commercially available transwell inserts to be nested in the wells of the upper layer, thus enabling the performance of a cell co-culture by seeding cells both on the upper surface of the porous membrane of the transwell and on the Gel-Pak membrane, which are separated by a distance of 3 mm. Therefore, cells cultured on the transwell insert could be exposed to either the same or different oxygen levels to those cells growing on the chip surface, by modulating the oxygen concentration in the gas mixture provided by the gas blender and oxygen levels in the cell culture incubator.

The setting was constructed using polylactic acid (PLA) (RS Components, Madrid, Spain) molds designed with Autodesk 123D Design software and printed with an Ultimaker-2 3D printer (Ultimaker, Geldermalsen, The Netherlands). The code driving the 3D printer is provided in Supplementary Materials. The molds were employed to fabricate PDMS layers via replica molding. Essentially, a PDMS mixture (Sylgard 184 kit, Dow Corning, MI) prepared as described before (12) was poured onto PLA molds, degassed in a vacuum desiccator (Kartell Labware, Noviglio, Italy) and cured in an oven (Selecta, Barcelona, Spain) at 65°C for 3 h. Then, PDMS replicas were bonded to the Gel-Pak membrane using plasma treatment as described previously (12). Subsequently, the central region of the assembled device was drilled to allow the passage of a tube to connect wells of the lower layer to the gas source. Finally, the base of the device was adhered into a 100 mm diameter culture dish (Techno Plastic Products AG) using PDMS and the lid was also drilled to enable the passage of the inlet tube. This fabrication process is illustrated by a video provided in the Supplementary Materials.

The actual values of oxygen partial pressure (PO_2_) at cell level (on top of the transwell insert and on top of the PDMS membrane) were measured in real time by using an O_2_ sensor (FireStingO_2_, PyroScience, Aachen, Germany), a fiber-optic oxygen meter (Figure 2).

### Experimental Groups

B16F10, E0771, RENCA, and RAW264.7 single cultures maintained in T-25 flasks were employed to perform the experiments. Immediately before tumor cell seeding, the cell culture devices were UV-sterilized, and membranes were surface-coated with proteins of the extracellular matrix as previously described (12). More specifically, 100 μg/ml type I collagen (Cultrex, Trevigen Inc., Gaithersburg, MD) or 10 μg/ml fibronectin were employed for E0771 and B16F10 cell cultures, respectively. Then, 1.5 ml of complete growth medium containing 65,000 B16F10, 75,000 E0771, or 80,000 RENCA cells were added to each well. Tumor cells were allowed to settle on the Gel-Pak membrane and maintained at 5% CO_2_ balanced-air, 37°C and 100% humidity in a standard cell culture incubator for 24 h. Then, commercial transwell inserts with 8 μm pore polycarbonate membrane (Corning Inc., NY) were nested in wells of the upper compartment. Subsequently, 150,000 RAW264.7 cells suspended in 0.3 ml of serum-deprived medium were added on top of the porous membrane. Then, the devices were placed in a cell culture incubator at 37°C and 100% humidity and connected to a gas source providing a gas mixture with either 20% O_2_ (normoxia) or 1% O_2_ (hypoxia) for 24 h. Tumor cells and macrophages were cultured under same (20% O_2_) (N-N) or different oxygen concentrations [1% O_2_ for tumor cells and 17% O_2_ (16–19%) for macrophages] (H-N) simulating the standard conditions in cell research or those conditions representative of the tumor-vessel interface, respectively. Cells cultured in each experiment were obtained from the trypsinization of different subcultures in order to perform six independent cell co-cultures per device. Experiments were performed in duplicate: one for the assessment of macrophage migration and tumor cell proliferation and the second to determine the M1/M2 polarity of macrophages (*n* = 6). Moreover, single cultures of tumor cells on the surface of the device were performed in duplicate in order to analyze potential changes in cancer cell proliferation and expression of genes involved in macrophage recruitment in response to oxygenation levels. Similarly, single cultures of macrophages on the upper side of the porous membrane were also employed as negative controls for the determination of macrophage migration.

### Assessment of Macrophage Migration

At the end point of experiments, transwell inserts were transferred to bottom plates containing 0.5 ml of 1X PBS supplemented with 2% FBS. Macrophages remaining on the upper side of the porous membrane were removed using cotton tipped applicators, and cells present in the lower side were fixed and stained with hematoxylin and eosin (Panreac AppliChem, Barcelona, Spain). Plates were placed on the stage of an inverted microscope (Zeiss Axiovert S100, Oberkochen, Germany) and bright field images of the lower face of the porous membrane were acquired using a uEye SE camera (IDS Imaging Development Systems Gmbh, Obersuln, Germany) and 5X magnification lens by an investigator who was blinded to the treatment group. The regions containing hematoxylin/esosin-stained cells were identified by an automated thresholding plugin in ImageJ software (13). For each image, cell migration was calculated as the quotient between the area of the transwell membrane covered by cells and the total area of the image and expressed as percent ( × 100). Cell migration was normalized to the control group (N-N).

### Assessment of M1/M2 Polarization of Macrophages

To determine whether oxygen concentrations could exert a potential effect on tumor cells affecting macrophage polarization, CD86 and CD206 surface markers representative for M1 (anti-tumoral) and M2 (pro-tumoral) macrophage phenotypes, respectively, were appraised using fluorescence activated cell sorting (FACS) analyses. In order to collect RAW264.7 cells remaining on both sides of the transwell membrane, the inserts were firstly rinsed with 1x PBS and then transferred into bottom plates containing 0.05% trypsin/0.02% ethylenediaminetetraacetic acid (Sigma-Aldrich). The obtained cells were stained with CD86-PerCP/Cy5.5 and CD206-APC fluorescence-conjugated antibodies and analyzed on a FACS Canto II cytometer using FACS Diva 5.5 software (BD Biosciences, San Jose, CA). Data were analyzed with FlowJo software (Tree Star, San Carlos, CA) to compute the median fluorescence intensities (MFI) of CD206 and CD86 markers.

### Tumor Cell Proliferation

Tumor cells adherent to the Gel-Pak membrane were trypsinized and labeled with CD45-FITC antibody to discard any macrophage (CD45+ cells) that potentially could detach and adhere to the tumor cell culture. In single cultures, the same protocol was followed although CD45 staining was not performed. Total number of tumor cells in the sample was represented by the number of unstained live cells identified by FACS.

### Determination of PTGS2, VEGFA, and EMAPII Gene Expression in Tumor Cells

At the end point of experiments, RNA was isolated from tumor cell single cultures using the RNeasy kit (Qiagen, Hilden, Germany) following manufacturers' instructions. Then, 0.5 μg of RNA were employed to synthetize cDNA by reverse transcription polymerase chain reaction (PCR; TaqMan Reverse Transcription Reagents). Changes in the expression of prostaglandin-endoperoxide synthase 2 (PTGS2), vascular endothelial growth factor (VEGFA), and endothelial-monocyte activating polypeptide II (EMAPII) genes were analyzed by quantitative PCR using the Taqman Fast Advanced Master Mix in a StepOne Plus thermocycler. Relative PTGS2, VEGFA, and EMAPII gene expression was normalized to the expression of peptidylprolyl isomerase A (PPIA) gene applying the 2^−ΔΔ*Ct*^ method (14) to compute the fold change in expression of these genes compared to baseline. Unless otherwise specified, all reagents were purchased from ThermoFisher Scientific (Waltham, MA).

### Statistical Analysis

Data are presented as means with standard error (SE) bars (*n* = 6). Differences in means between N-N and H-N groups were analyzed by two-tailed *t*-tests (when applicable) or by the Mann-Whitney test. Tumor cell proliferation was compared by two-way analysis of variance (ANOVA) with type of culture (single culture vs. co-culture) and gas condition (N-N vs. H-N) as variables. Student-Newman-Keuls *post hoc* method was employed for multiple comparisons. All statistical analyses were performed with SigmaPlot (Systat Software, San Jose, CA) and *p* < 0.05 were considered as statistically significant.

## Results

### Generation of a Differential Oxygen Microenvironment in the Co-culture Device

The wells of the novel cell device allowed the introduction of commercially available transwell inserts to recreate a co-culture of different cells with each simultaneously exposed to different oxygen level (Figure 2A). Specifically, the cells cultured on the conventional transwell insert can be exposed to either the same or different oxygen levels to those cells growing on the chip surface by modulating the oxygen concentration in the gas mixture provided by the gas blender and oxygen levels in the cell culture incubator. We set oxygen gradient to mimic *in vivo* conditions (Figure 2B). Three types of tumor cells where subjected to continuous hypoxia (1% O_2_) and macrophages where maintained to 16–19% O_2_ in co-culture (Figure 2C), as determined by a fiber-optic oxygen sensor. This setting allowed us to study the activation and migration of oxygenated macrophages in response to hypoxic tumor cells.

### Melanoma and Breast Tumor Cells Exposed to Hypoxia Enhance Macrophage Migration

**Figure 3:**
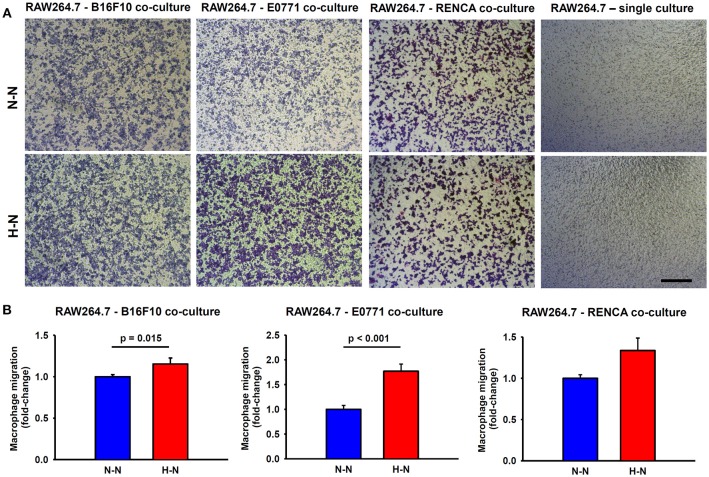
Macrophage migration through a permeable membrane in co-culture with mouse melanoma (B16F10), breast (E0771), or renal (RENCA) cancer cells exposed to different oxygen levels. **(A)** Representative bright field images showing stained macrophages which migrated to the lower side of the transwell membrane after 24 h in co-culture with melanoma, breast, or renal cancer cells cultured under normoxic conditions (N-N) or under a physiological gradient of oxygen (H-N). In the absence of tumor cells (right), macrophages did not migrate through the transwell membrane. Scale bar = 250 μm. **(B)** The number of macrophages (RAW 264.7) that migrated to the lower side of the membrane was significantly higher when B16F10 (left) or E0771 (center) were selectively exposed to hypoxia (H-N) in comparison to normoxia (N-N), while no differences were found between treatments in the migration of macrophages co-cultured with RENCA cells (right). Data were normalized to the control group (20% O_2_).

As shown in Figure 3, tumor cells are essential to promote macrophage migration to the lower side of the porous membrane. When macrophages were in co-culture with either melanoma or breast cancer cells exposed to hypoxia (hypoxia-normoxia; H-N) their migration increased by ~15% (*p* = 0.015) and ~70% (*p* < 0.001), respectively, in comparison to 20% O_2_ (normoxia-normoxia; N-N). Although RENCA cells also promoted the migration of RAW264.7 cells to the lower side of the transwell membrane, no significant changes were observed in response to the different experimental conditions (N-N and H-N). In the absence of tumor cells, the differential oxygen concentration did not have any effect on macrophage migration when comparing N-N and H-N groups (Figure 3A, right). Therefore, macrophage migration through the porous membrane was induced through soluble mediators produced by tumor cells, rather than by the differences in the oxygen levels between the upper and bottom compartments of the migration assay system.

### Hypoxia-Treated Melanoma and Breast Tumor Cells Reduce M1 Expression in Macrophages

**Figure 4:**
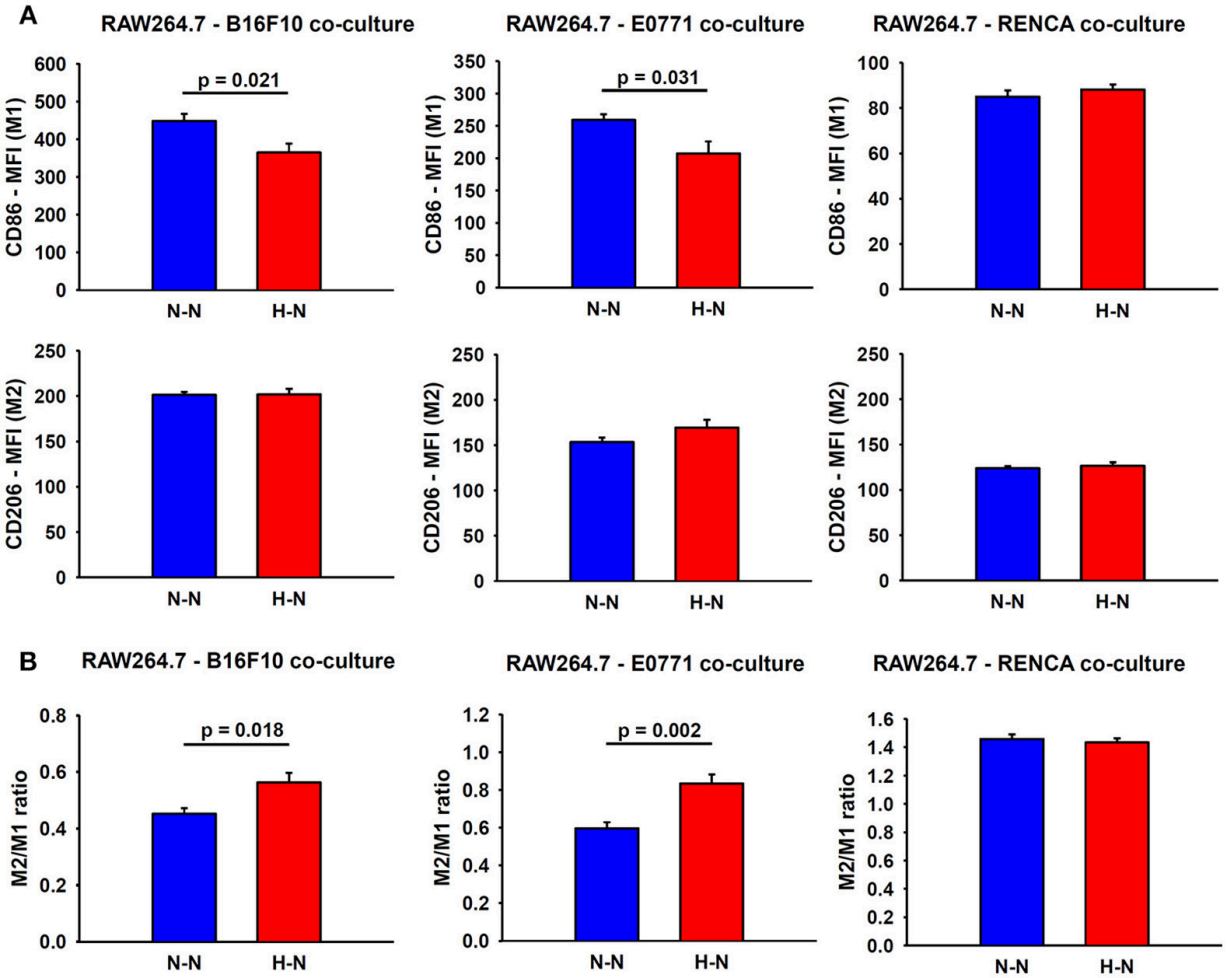
Assessment of macrophage polarity. **(A)** Representative graphs obtained during FACS analyses which depict the median fluorescence intensities (MFI) for M1 and M2 markers in macrophages co-cultured with melanoma (left), breast (center) and renal (right) cancer cells exposed to hypoxia (H-N) or normoxia (N-N). Hypoxia-treated melanoma and breast tumor cells reduced the M1-like polarization of macrophages **(A)**, as reflected by an increase in the M2/M1 ratio compared to that observed when both cell types were exposed to normoxia (N-N) No significant changes in the phenotype of macrophages were observed when co-cultured with renal cancer cells exposed to different oxygen levels **(B)**. The M2/M1 ratio was calculated as the ratio of the median fluorescence intensity (MFI) of CD206/CD86 surface markers.

In addition to an increased migratory capacity, macrophages in co-culture with melanoma or breast cancer cells exposed to hypoxia (H-N) showed a marked reduction in the expression of CD86 (M1 marker) (19% ± 5; *p* = 0.021 and 20% ± 7; *p* = 0.031, respectively) when compared to normoxic oxygenation in both compartments (N-N). In contrast, no significant changes were observed in the expression of CD86 in macrophages co-cultured with hypoxia-treated renal cancer cells respect to those exposed to normoxia. In general, no differences in the CD206 marker were observed between groups. The resulting M2/M1 MFI ratio obtained in H-N conditions was also increased when macrophages were co-cultured with melanoma (*p* = 0.018) and breast cancer cells (*p* = 0.002) as compared with N-N (Figure 4).

### Hypoxia-Induced Melanoma Cell Proliferation Is Further Increased in Co-culture With Macrophages

**Figure 5:**
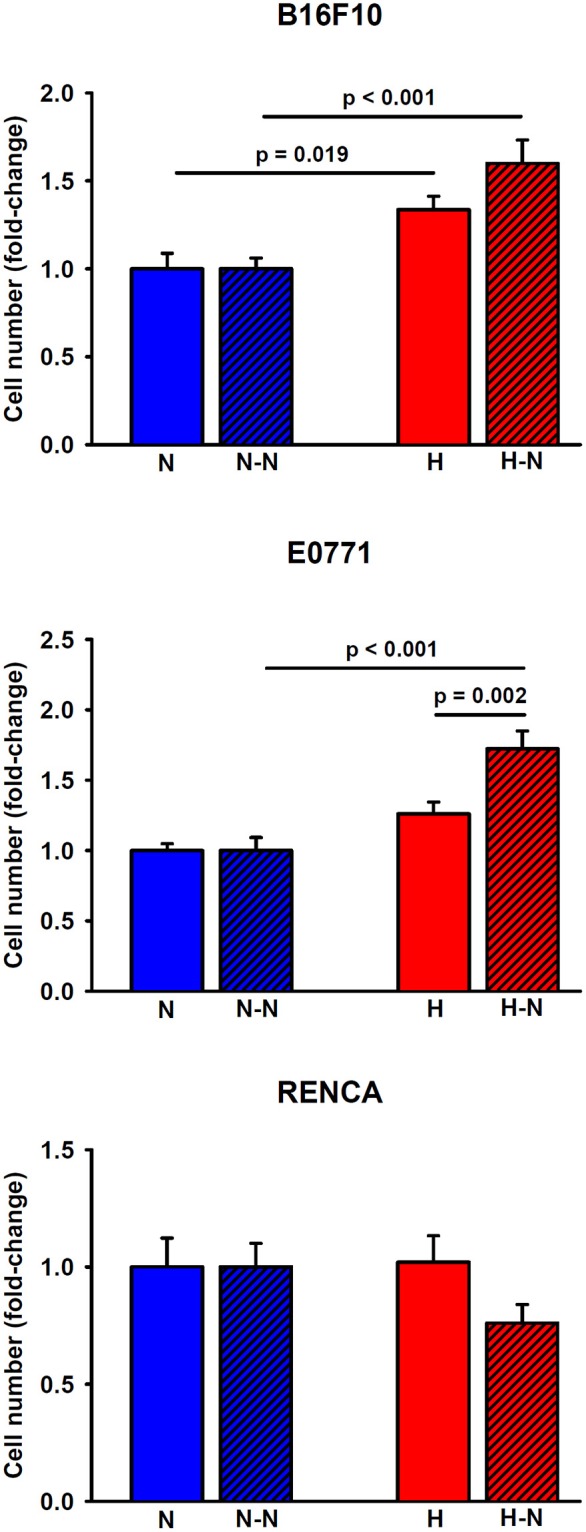
Proliferation of melanoma, breast, and renal cancer cells. Melanoma (upper) and breast (middle) cancer cells increased their proliferative rates when cultured under low levels of oxygen either in single culture (H) or in co-culture with normoxic macrophages (H-N) when compared to N and (N-N) treatment groups, respectively. The hypoxia-induced proliferation observed in single culture (H) was further enhanced when tumor cells were co-cultured with normoxic macrophages (H-N). Renal cancer cell proliferation remained similar among the different treatment groups (right).

As shown in Figure 5, the application of hypoxia (1% O_2_) to melanoma cells in single culture for 24 h increased their proliferation by 34 ± 8%, (*p* = 0.019) in comparison to the corresponding values observed at 20% O_2_. Similarly, hypoxic conditions promoted a trend toward an increased breast tumor cell proliferation (26 ± 8%, *p* = 0.060) compared to normoxia. Notwithstanding, renal cancer cell proliferation was not affected by oxygenation levels.

When melanoma or breast cancer cells were selectively subjected to hypoxia in co-culture with macrophages (H-N), they exhibited increased proliferation (60 ± 13%, *p* < 0.001 and 72 ± 13%, *p* < 0.001, respectively) when compared to the N-N conditions. The increased proliferation under H-N conditions was more pronounced in co-culture for breast cancer cells (*p* = 0.002) when compared to single culture under hypoxia (H). A similar trend, although no significant, *p* = 0.058), was observed in co-cultures of melanoma cells (H-N) when compared with single cultures (H). No significant differences in renal cancer cell proliferation were observed in response to different levels of oxygenation regardless of the presence or absence of macrophages. Therefore, these findings suggest the existence of a positive feedback loop between breast and melanoma tumor cell proliferation and increased migration of macrophages presenting an increased M2/M1 ratio when subjected to differential oxygenation levels as traditionally encountered in solid tumors.

### Alterations in PTGS2, VEGFA, and EMAPII Genes in Tumor Cells Following Hypoxia Treatment

**Figure 6:**
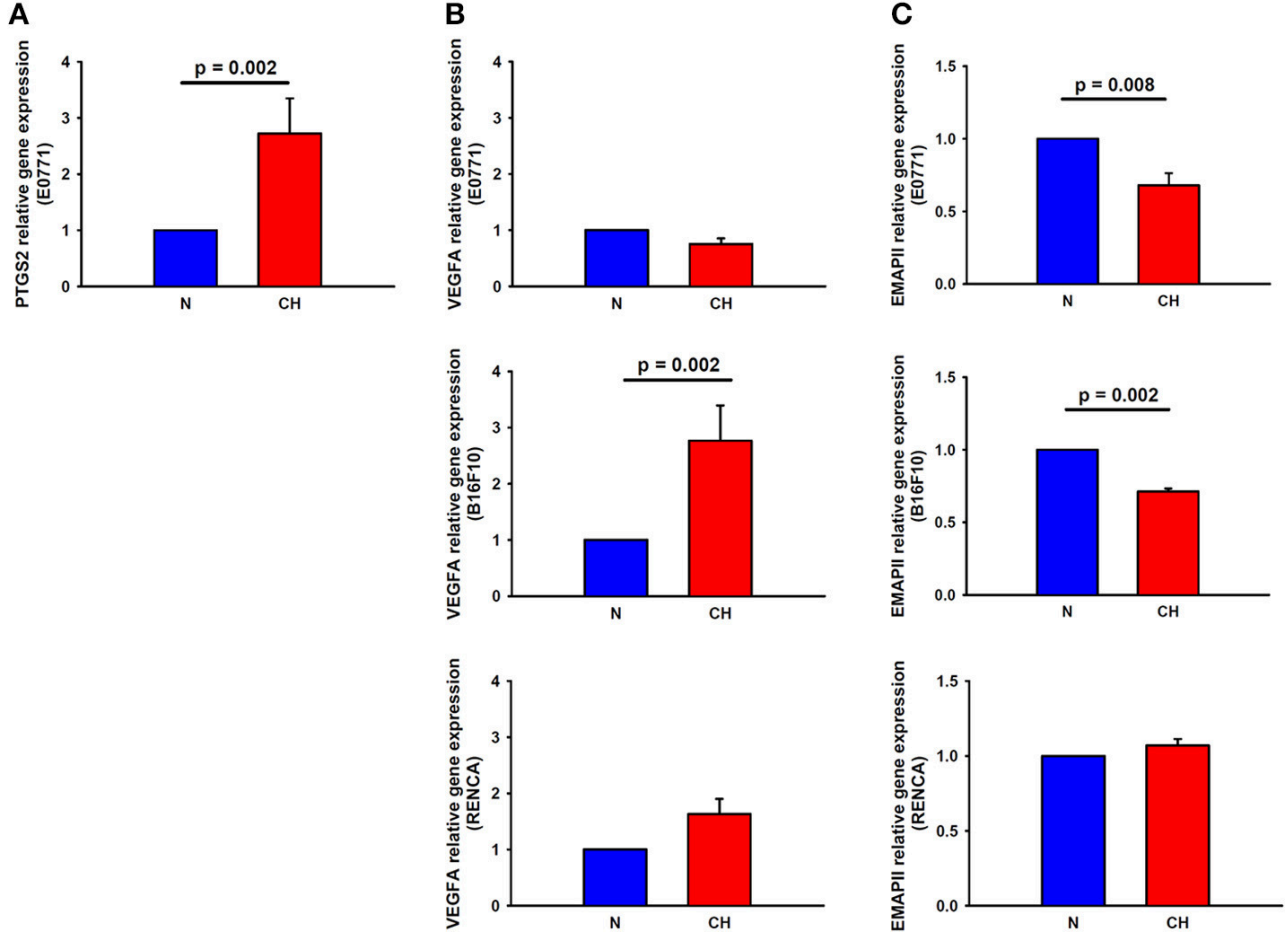
Expression of macrophage recruitment-related genes in melanoma, breast, and cancer cells in response to hypoxia. PTGS2 **(A)** and VEGFA (**B**, center) gene expression was upregulated in hypoxia-treated breast and melanoma cancer cells, respectively, in comparison to normoxia. **(C)** EMAPII gene expression was reduced by hypoxia in breast (upper) and melanoma (center) tumor cells, while remained invariable in renal cancer cells (lower).

PTGS2 gene expression experienced a 2.7-fold increase in breast cancer cells cultured under hypoxic conditions for 24 h when compared to normoxic conditions (*p* = 0.002), suggesting a role for this factor in the higher macrophage recruitment observed in H-N co-culture (Figure 6A). Nonetheless, the expression of this gene was undetectable by real-time polymerase chain reaction in melanoma and renal cancer cell cultures, suggesting that other genes rather than this one would be involved in the highest recruitment of macrophages promoted by hypoxia-treated melanoma cells. In this sense, a 2.7-fold increase (*p* = 0.002) in the expression of VEGFA in B16F10 cells was detected in response to hypoxia (Figure 6B). Regarding the expression of EMAPII, it was lower in hypoxia-treated breast and melanoma tumor cells and remained similar between H-N and N-N conditions for renal tumor cells (Figure 6C).

## Discussion

The novel cell culture setting we developed enables improved understanding of the processes leading to activation and recruitment of host immune cells during tumor development. In spite that tumor hypoxia has been widely suggested as a main hallmark of solid cancers, the oxygen gradients experienced by the different types of cells playing a role in tumors have not been recreated in most experimental research because it was not easy to mimic with the conventional *in vitro* settings available. The practical approach presented here provides an easy way to reproduce *in vivo* oxygen gradients and hence to study the crosstalk between tumor cells and macrophages in the presence of differential oxygen levels. As a proof-of-concept of the potential usefulness of the setting, our results provide support to the notion that divergent oxygenation patterns across a gradient as observed in solid tumors play an important role in the interaction between tumor cells and host immune cells. Interestingly, we observed that the effects of a hypoxic gradient between oxygenated macrophages and hypoxic cancer cells on macrophage activation, recruitment and cancer cell proliferation were cell type dependent, and most importantly were different to those found when using conventional Boyden chamber migration assays (where both cell types exposed to the same oxygen levels).

It is now well-recognized that tumor associated macrophages are key drivers of cancer progression, facilitating tumor growth, invasiveness and neoplastic transformation (7, 9, 10, 15, 16). Resident macrophages, which are distributed throughout every organ of the body, or bone-marrow derived monocytes are massively recruited from the circulation to the tumor microenvironment in response to chemoattractant molecules secreted by cells within the tumor microenvironment (7, 15, 17). To study the crosstalk of tumor cells with macrophages, different adaptations of the original Boyden chamber assays have been employed and have provided critical insights on the potential mechanisms involved in such crosstalk (18–21). By using this approach, a vast number of studies have assessed the migration, invasion, and metastatic potential of tumor cells in co-culture with macrophages (18, 20, 21).

Despite tumor hypoxia is raising great interest due to the increased evidence linking low levels of oxygen with a more aggressive tumoral phenotype and higher tumor resistance to different forms of therapy (2, 4, 22–24), the number of studies including hypoxia as a modifiable variable in the assessment of interactions between cancer cells and macrophages and their recruitment to the tumor is very scarce. Some of the available studies have applied either hypoxia or normoxia to the cell co-culture employing different modifications of the Boyden chamber in order to compare the motility and/or invasiveness of tumor cells and/or macrophages in different oxygen environments (25). In a similar fashion, other investigators have pre-exposed tumor cells and/or macrophages to hypoxia, and subsequently, the hypoxic cells or supernatants were employed to test their effects on macrophages and/or tumor cell malignancy (10, 18, 20). However, both of such abovementioned approaches have notable limitations. For example, to study the mechanisms involved in the recruitment of macrophages to the hypoxic tumor mass, Triphati et al. introduced macrophages in the upper well of a modified Boyden chamber, while 24 h pre-exposed hypoxic/normoxic breast cancer cells or conditioned media were positioned into the lower wells (10). These authors observed a markedly increased migration of macrophages in response to breast cancer cells pre-exposed to hypoxia and conditioned media. Thus, the tumor cells were not actually exposed to hypoxia during their co-culture with macrophages, while no crosstalk between cells occurred when conditioned media was used. However, when the Boyden chamber was subjected to hypoxia (i.e., both macrophages and tumor cells were then simultaneously exposed to hypoxia), the authors observed that the migration was reduced compared to the findings observed when breast cancer cells were pre-exposed to hypoxia. Therefore, we interpret these studies as indirectly suggesting that the different oxygenation levels that are present in cancer cells and circulating macrophages could foster the directional migration and recruitment of macrophages into the tumor stroma. Alternatively, some investigators have designed 3D culture systems to create a hypoxic gradient from the periphery to the core of the sphere in an attempt to recreate a “solid tumor sphere” (26, 27). Despite the fact that this innovative methodology is able to generate an oxygen gradient, the oxygen gradient itself is not well-controlled, and is potentially dependent on the metabolic rate of the cancer cells and the size of the sphere, thereby creating inordinate variability from one experiment to the next (27). Furthermore, these 3D cultures do not allow for the concurrent culture of two cell populations separated at different oxygenation levels, a desirable situation when studying the potential mechanisms responsible for cancer cell invasion induced by macrophages. In the present study, we have implemented a new setting that is capable of concurrently subjecting two cell types to different oxygen levels mimicking the tumor microenvironment. This novel approach allowed us to evaluate changes in macrophage migration induced by tumor cells when the latter were selectively exposed to hypoxia in co-culture. Furthermore, in contrast to previously reported microfluidic devices capable of generating different oxygenation environments (28–31), the macroscale experimental setting presented herein is easily implementable in most laboratories, given its easy fabrication, low-cost, and expanded versatility. In the present work, melanoma and breast cancer cells promoted macrophage migration *in vitro* as previously reported for other types of cancer cells (10, 32). Indeed, macrophages cultured in the upper surface of the porous membrane in the absence of tumor cells being concurrently cultured in the bottom compartment did not migrate in either one of the two experimental groups (N-N and H-N), thereby confirming that migration of macrophages is induced by soluble factors being released by cancer cells rather than by the oxygen gradient *per se*.

Two major macrophage phenotypes have been described, namely the classically activated (M1) and alternatively activated (M2) macrophages. These macrophages can be identified based on their distinctive expression of several surface markers and cytokine release (33). Concretely, M1 macrophages mainly express a series of pro-inflammatory cytokines leading to effective tumoricidal properties, while M2 macrophages, which secrete anti-inflammatory cytokines along with other angiogenic molecules and factors suppressing inflammation, facilitate tumor cell immune evasion and survival (34, 35). Based on the observation that tumor cells can re-educate macrophages toward an alternative phenotype, cancer treatments that are predicated on immunotherapy are being increasingly designed to boost the immune defenses against cancer, by either maintaining or promoting the M1 macrophage phenotype (34, 36). We should also remark that M2 macrophages can directly promote cancer cell proliferation, migration and metastatic potential (37, 38). In fact, tumor cells induce a shift toward M2 phenotype in isolated macrophages and that such shift will be further accelerated under exposures to sustained or cyclical hypoxia (15, 39). Here, we have found that hypoxia increased the proliferation of melanoma and breast cancer cells and, in addition, the crosstalk between hypoxic tumor cells and normoxic macrophages (H-N) promoted macrophage recruitment and reduced their polarization toward a M1 phenotype and consequently increased their M2/M1 ratio in a similar fashion to our previous findings from tumor associated macrophages isolated from tumors of mice exposed to intermittent hypoxia (IH) (39) and explaining the higher recruitment of macrophages in the tumor microenvironment (15). Moreover, the increased recruitment of these macrophages by hypoxic tumor cells promoted further increases in their proliferation, demonstrating the presence of an intensified feedback loop when an oxygen gradient is applied. These findings are in keeping with previous *in vitro and in vivo* reports showing that migrated macrophages in response to pre-exposed breast and melanoma cancer cells to hypoxia presented a marked polarization toward the M2 phenotype (10, 40). Furthermore, the exposure of melanoma and breast cancer cells to hypoxia resulted in their increased proliferation as previously reported (39, 41). On the other hand, renal cancer cell proliferation was not altered by oxygenation levels and macrophage recruitment and polarization remained similar between the different conditions. These results are in keeping with previous *in vivo* observations from RENCA subcutaneous tumors in Balb/c mice subjected to intermittent hypoxia compared to room air control (42). Application of IH was not able to promote a pro-tumoral phenotype in tumor-associated macrophages and no changes in tumor growth or metastasis were found. Also, VEGFA gene expression by RENCA cells was also not affected by IH *in vitro* (42). Therefore, the differential response to the hypoxic stimulus assessed in this study suggests that the effect of this stimulus is highly cell type dependent and needs to be analyzed for each specific cancer.

The model employed also showed that melanoma and breast cancer cells attracted macrophages through paracrine signaling, since no contact was present between the tumor cells and macrophages. It has been widely described that hypoxia inducible factor-1 (HIF-1) and nuclear factor-kappa B (NF-κB) are activated in response to hypoxia (43–45), thus modulating the expression of genes such as VEGFA, cyclooxygenase 2 (COX-2) and those encoding for a wide array of cytokines and chemokines including EMAPII, endothelin-2 (EDN2) or the monocyte chemoattractant protein 1 (MCP1), which have been distinctly recognized as playing important roles in the recruitment, polarization and survival of macrophages in tumors (46–50). Other studies have reported that the overexpression of PTGS2 and the accumulation of its by-product prostaglandin E2 (PGE_2_) within hypoxic regions of several types of tumors are associated with the increased recruitment and M2-like polarization of macrophages that lead to poor cancer outcomes (51). In fact, the treatment of celecoxib, a specific inhibitor of COX-2, was able to suppress the IH-induced tumor growth and macrophage activation (52). Similarly, VEGF can be also released in response to hypoxia by both tumor and tumor stroma cells, acting not only as a regulator and promoter of angiogenesis, but also as a chemoattractant for macrophages (47, 53). In this line, EMAPII protein levels also increased in several cancer types including melanoma, fibrosarcoma, colorectal and hepatocellular carcinomas in response to hypoxia both *in vitro* and *in vivo* (48, 54, 55). In the present study, qPCR analyses revealed a higher expression of PTGS2 or VEGFA genes in hypoxia-treated breast and melanoma cells, respectively. These results suggest that, although PGE_2_ and VEGF are likely involved in the reported changes in macrophage phenotype and their increased recruitment in the presence of an oxygen gradient, their activation seems to be tumor cell-specific. Regarding EMAPII, mRNA levels were significantly lower in hypoxia-treated melanoma and breast cancer cells as compared with normoxic conditions. Nonetheless, the activation of EMAPII by hypoxia remains controversial: although the upregulation of this factor in response to hypoxia has been usually described, there is a study that reports its downregulation in hypoxia-treated uveal melanoma cells (48) and, in addition, other studies have reported that the regulation of this mediator by hypoxia is more likely exerted at the translational or post-translational level (56). Regarding renal cancer cells, the existence of the same hypoxic gradient did not exert any effect on the expression of VEGFA and EMAPII genes, and therefore, in the concomitant macrophage migration and polarization. For all these reasons, drawing any clear conclusion about the exact molecular mechanisms involved in the recruitment and polarization of macrophages requires comprehensive studies including gene and protein arrays for each specific tumor type.

In summary, the novel experimental setting described and tested here is capable to maintain cells in co-culture and to simultaneously expose each cell type to different oxygenation. This experimental setting can be easily built by any conventional 3D printer using non-expensive materials thereby it can be implemented by any laboratory investigating cell tumors under realistic oxygenation microenvironments. We carried out proof-of-concept experiments to illustrate the feasibility of the new setting and to proof that oxygen gradients result in results different from the ones obtained using the conventional setting which is unable to recreate realistic gas partial pressure gradients. Specifically, we have shown that oxygen gradients play a role in modulating macrophage recruitment to the tumor, via the promotion of increased macrophage migration and accelerated polarization toward the M2 phenotypes, which in turn leads to increased cancer cell proliferation. Interestingly, the response induced by the hypoxic gradient is also cancer type dependent indicating that some tumors could be more sensitive to hypoxia. Our experiments were not addressed to investigate in detail the mechanism involved and therefore future research mimicking differences in cell oxygenation are required to improve our understanding of the mechanisms involved in the cross-talk between tumor cells and other host cells such as fibroblasts, immune cells, stem cells and adipocytes can occur. From a practical perspective, it is noteworthy that the novel experimental setting described here can be easily adapted to other types of commercially available assays to study the interaction, migration, invasion, and/or endothelial intra/extravasation of different types of tumor stromal cells while they are exposed in physiologically relevant, yet different oxygen partial pressure conditions. Since the new experimental setting is also capable of reproducing the cyclic hypoxia that is encountered in the tumor during aberrant blood flow (12), it offers unique research opportunities in the exploration of pathophysiological mechanisms and therapeutic targeting focused on tumor hypoxia.

## Author Contributions

NC performed experiments, analyzed data, and drafted components of the manuscript. BF, JO, and RC performed experiments, analyzed data, and served as blinded observers. DG, DN, and RF provided critical insights and participated in the drafting of the manuscript. IA conceptualized the project, analyzed data, and drafted the previous versions of the manuscript. IA, RF, and DN are responsible for the financial support of the project. All authors have reviewed and approved the final version of the manuscript.

### Conflict of Interest Statement

The authors declare that the research was conducted in the absence of any commercial or financial relationships that could be construed as a potential conflict of interest.
